# Nonsynostotic plagiocephaly: a child health care intervention in Skaraborg, Sweden

**DOI:** 10.1186/s12887-019-1405-y

**Published:** 2019-02-06

**Authors:** Freda Lennartsson, Per Nordin

**Affiliations:** 1 Department of Pediatrics, The Sahlgrenska Academy, University of Gothenburg, 416 85 Gothenburg, Sweden; 2 The Skaraborg Institute for Research and Development, Stationsgatan 12, 541 30 Skövde, Sweden

**Keywords:** Assessments, Brachycephaly, Intervention, Nonsynostotic plagiocephaly, Prevention, Reversal

## Abstract

**Background:**

The aim was to evaluate the intervention’s effect on prevention and reversal of nonsynostotic plagiocephaly.

**Methods:**

Thirty-eight intervention group nurses were educated about nonsynostotic plagiocephaly and asked to follow guidelines; 18 control group nurses were not. In a longitudinal single-blinded clinical intervention, parents brought infants to well-child visits according to the national schedule. Cranial shape was assessed in 176 intervention and 92 control group infants at 2-, 4-, and 12-month visits.

**Results:**

Asymmetry at two months reversed by four months four times more often in intervention than control subgroup infants (OR = 4.07, *p* = 0.02) when adjusted for parent awareness of written information from their nurse. Asymmetry at two months reversed by 12 months fivefold when parents were aware of written information (OR = 0.19, *p* = 0.04). The risk for persistent asymmetry at 12 months was lower for intervention than control group infants (RR = 0.35, *p* = 0.03). Of infants with no asymmetry at two months, 25% in intervention and 22% in control group developed brachycephaly.

**Conclusions:**

The intervention contributed to early reversal and reducing infants’ risk for persistent asymmetry. Parents’ awareness of written information contributed to reversal. Preventing brachycephaly was difficult. Further research is needed.

**Electronic supplementary material:**

The online version of this article (10.1186/s12887-019-1405-y) contains supplementary material, which is available to authorized users.

## Background

Nonsynostotic plagiocephaly (NSP) is acquired cranial asymmetry resulting from physical forces applied over a time, and refers to altered cranial shape in infants older than six weeks of age, when molding from the birth process is over [1]. Nonsynostotic plagiocephaly falls into three main groups: plagiocephaly - skewed occipital flattening, brachycephaly - symmetric occipital flattening, and combined plagiocephaly/brachycephaly [2]. The most commonly reported risk factors are: first-born, male, limited neck rotation or preference in head position, supine sleep position, lower level of activity, and lack of tummy time [3]. An increase in prevalence of NSP was noted in American tertiary centers the 1990s, and this was largely attributed to parents following the recommendation to place their infant supine while sleeping in order to prevent sudden infant death (SIDS) [4]. In a prospective cohort study from 2014, 47% of 440 healthy full-term infants seven to 12 weeks of age in Calgary were estimated to have NSP [5]. In a prospective cohort study investigating the natural course, the prevalence of NSP increased to four months, and the majority of cases reversed by two years of age [6]. Although NSP might disappear as a child grows older and increased mobility relieves pressure on the cranium, it persists in some children. In a study of 129 children diagnosed with NSP in infancy and whose parents had been given information on counter-positioning strategies, 39% had not reverted to the normal range of symmetry at mean age of four years [7].

Few studies have evaluated the effect of early intervention. In a prospective controlled study, there was a significantly lower prevalence of plagiocephaly at four months when parents received recommendations to encourage spontaneous unhindered physical movement within 72 h postpartum in addition to the usual infant positioning recommendations [8]. In a randomized controlled trial (RTC), researchers reported that educating parents on unhindered physical movement within 72 h postpartum in addition to regular recommendations significantly reduced the prevalence and severity of NSP at three months [9].

A project was initiated in Skaraborg in 2008 in an attempt to prevent NSP via child health nurses working within the Swedish National Child Health Care Program. The nurses are pediatric or public health nurse specialists. They are the primary health care providers responsible for monitoring infants’ growth and development and informing parents about the Swedish Board of Health and Welfare’s recommendations. Nearly all infants in Sweden attend the child health clinics, providing an ideal venue for early intervention. Guidelines for child health nurses were developed [10], tested in a pilot study [11], and then revised. A continuing education which included the guidelines was subsequently developed for the nurses [12]. The education included knowledge on how NSP develops, risk factors, which infants are extra vulnerable, how to assess cranial form, how to differentiate NSP from craniosynostosis, prevention recommendations for parents of newborns, and recommendations for parents on how to reverse incipient asymmetry [13]. The aim of this study was to evaluate the effect of the intervention on prevention and reversal of NSP. The assumption was that most NSP can be prevented if child health nurses are educated about NSP and provide parents of infants with early and on-going tailored counseling, and if parents in turn implement recommendations in their infant care. The research questions were: Did the intervention have an effect in preventing NSP? Did the intervention have an effect in reversing incipient NSP?

## Methods

### Setting, participants, and group allocation

The intervention was conducted at twenty-six child health centers in Skaraborg. The clinics served similar populations. Participants included 35 intervention group and 18 control group nurses, 182 intervention group and 92 control group infants, and the infants’ parents. Mean infant weight was 3610 in the intervention group and 3651 in the control group. Nurses were allocated into two groups in order to avoid the so-called spill-over effect where nurses with previous exposure to the project might influence and bias colleagues who had no previous exposure to the project. Thus, if any nurse at a clinic had participated in the pilot study and/or had attended the lecture on NSP held in December 2010, all nurses at that clinic were assigned to the intervention group. Nurses at clinics where no one had participated in the pilot study or attended the above-mentioned lecture were placed in the control group. Infants and parents were assigned to the same group as their nurse. As a result, the ratio of intervention group and control group infants introduced into the design of the study was 2:1. The estimated sample size needed was 160 intervention group and 80 control group infants, when taking into account a 95% confidence interval, a 90% power, and an estimated effect size of 17% derived from the pilot study [11]. Nurses in both groups recruited infants to the study in February 2012, and this date was extended until there were enough infants in the sample. Intervention group infants received care from nurses who had participated in the continuing education and control group infants received routine care. 275 infants were eligible for the study. One infant’s parents did not consent to participation. Drop-out included three (6%) nurses who terminated employment and six (2%) infants and their parents that moved (Additional file 1: Figure S1 and Additional file 2: Figure S2 in Additional files).

### Design

A longitudinal single-blinded clinical intervention with two arms was initiated in January 2012. First, the project leader educated intervention group nurses about NSP at their workplace in one-to-one or small group sessions which took about 1½ hours, and instructed them to work according to guidelines they were given. They were asked to assess cranial form weekly during the first months and then monthly until six months of age; to begin informing newborns’ parents about NSP prevention at the first home visit - which takes place when infants are about one week old - or at least by the time infants are two weeks old; and to encourage parents to begin getting their infants accustomed to tummy time by two weeks of age. Intervening when infants are one to two weeks of age is considered early intervention in this study because this is feasible within the child health program. Control group nurses did not participate in the education and were not asked to work differently. Secondly, nurses in both groups recruited infants by informing parents of newborns about the study and procured written informed parental consent. Parents brought their infants to well-child visits according to the national program’s schedule, which includes frequent visits during the first two months, then monthly visits until six months of age, and from then bi-monthly visits until 12 months of age.

### Data collection and materials

The project leader taught five individuals not employed at the clinics, four nurses and one medical secretary, how to assess infant cranial asymmetry using Severity Assessment for Plagiocephaly and Severity Assessment for Brachycephaly [14, 15] and then reliability-tested them [16]. These assessors were blinded to group assignment and specifically instructed to keep themselves uninformed about group assignment. They did cranial asymmetry assessments in conjunction with infants’ 2-month (T1), 4-month (T2), and 12-month (T3) well-child visits. Two nurses not employed at the clinics, one previous assessor and one nurse newly recruited to the study, were taught to take cranial measurements using a craniometer (Infocefalia, Barcelona, Spain). They were blinded to group assignment and took four cranial measurements in conjunction with 12-month well-child visits, cranial length, cranial width, and the two transcranial diagonals. Data were collected between March 2012 when the infants born first attended their 2-month well-child visit and October 2013 when the infants born last attended their 12-month well-child visit. Data regarding infant characteristics, birth-related factors, and infant care factors were collected by asking parents to fill in a form in conjunction with 2-month assessments. Data regarding information parents had received from their nurses were collected in a parent survey in conjunction with 4-month assessments [13].

### Assessment tools

Severity Assessment for Plagiocephaly consists of five sets of four-picture series, and Severity Assessment for Brachycephaly consists of three sets of four-picture series. Six of the eight picture sets were used in the study. Picture sets on “head tilt” and “facial asymmetry” from Severity Assessment for Plagiocephaly were left out because they do not depict cranial shape. Scores from 0 to 3 are attached to the four pictures in each set, where 0 designates no asymmetry, 1 designates mild, 2 designates moderate, and 3 designates severe asymmetry. We assume the difference between no asymmetry and mild asymmetry represents a small difference, while the difference between mild and moderate is an important difference, because moderate asymmetry includes skull base involvement and secondary asymmetries [17, 18]. We also assume that the asymmetry progression between moderate and severe is probably much more important, because as we see it, increasing cranial alterations involve increasing consequences for the infant [16]. We chose moderate asymmetry as the threshold for NSP since we consider asymmetry progression non-linear and since moderate asymmetry is the point where the skull base becomes involved.

### Rating system

Assessment scores from the picture sets that most specifically depict cranial shape were chosen to construct a rating system. The plagiocephaly score was derived from “posterior flattening” and “ear involvement” assessment scores, whichever was greater, to increase sensitivity. The brachycephaly score was derived from “posterior flattening” and “profile view” assessment scores, whichever was greater.

### Reliability

Cranial measurements were taken in order to reliability-test 12-month cranial assessments since Severity Assessment picture series have subjective components that increase the variation. Cranial vault asymmetry index (CVAI) was calculated from the two transcranial diagonals, and cranial index (CI) was calculated from the cranial length and cranial width measurements. The values were matched to standards for sex and age in order to arrive at cranial measurement scores [2] (Additional file 3: Table S1). Agreement between assessment scores and measurement scores at 12 months was analyzed. Measurement scores were considered the gold standard. The inter-rater agreement between 12-month assessment scores and measurements scores was analyzed using Agreement Coefficient 2 (AC2) with quadratic weights to reflect our assumption on the non-linear severity across the four-picture series’ “scale”. We adjusted agreement coefficients for chance agreement according to Gwet’s model to avoid inflating results [19], and then interpreted adjusted coefficients according to Landis and Koch’s intervals for strength of agreement for kappa statistics [20].

### Analyzing prevention and reversal

**Fig. 1:**
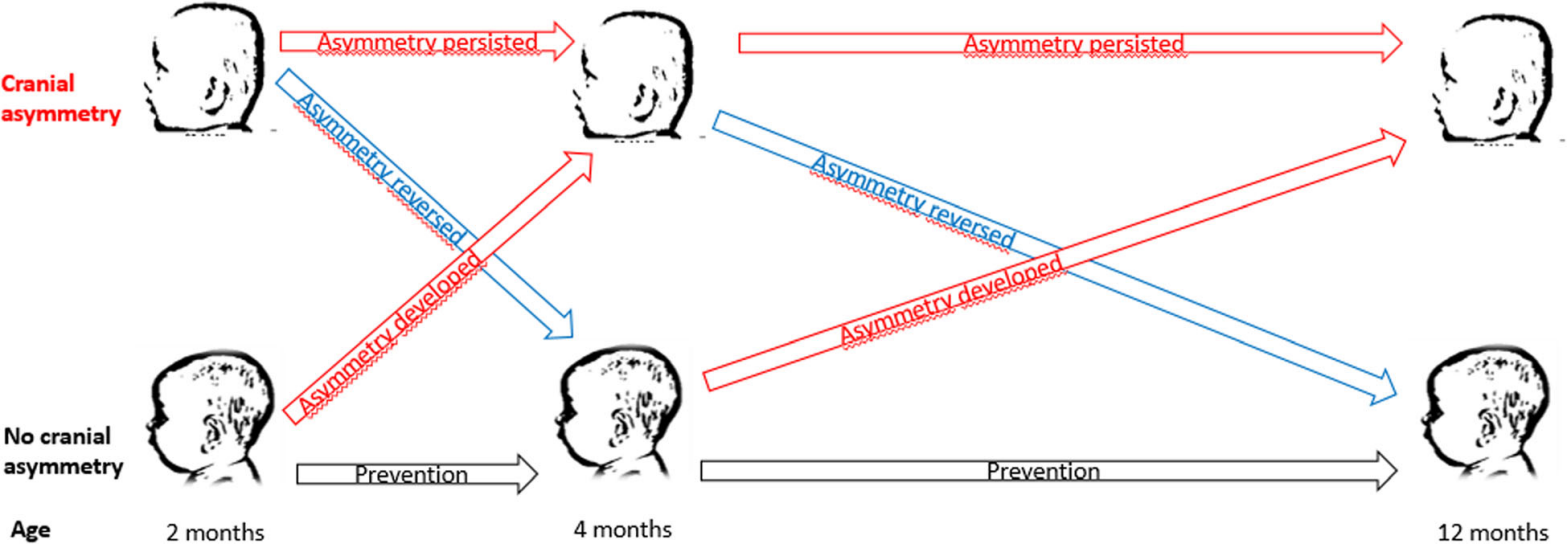
The ongoing processes of cranial asymmetry prevention and reversal in infants**.** Legend: Given a symmetric cranium, asymmetry can be prevented between assessments or asymmetry can develop – prevention failure. Given an asymmetric cranium, asymmetry can reverse between assessments or asymmetry can persist - reversal failure

Given a symmetric cranium, asymmetry can be prevented between assessments or asymmetry can develop - prevention failure. Given an asymmetric cranium, asymmetry can reverse between assessments or asymmetry can persist - reversal failure (Fig. 1). These are ongoing processes which can be influenced. The natural course, parents’ habits, and national recommendations conceivably influence cranial shape of infants. However, it is difficult to separate these influences.

As a tool for evaluating the effect of the intervention in terms of NSP prevention and reversal, we therefore chose to analyze the *change* that occurred between assessments, i.e., prevention failure and reversal. The occurrence of change is something that can easily be detected. We believe the occurrences of change are sufficient for making proper comparisons between the intervention and control groups. General NSP and the three main groups – plagiocephaly, brachycephaly and combined plagiocephaly-brachycephaly were analyzed in terms of prevention failure and reversal.

### Statistical methods

Descriptive statistics included: frequencies, percentages, medians, minimums-maximums, and graphs. Statistical analysis included: Chi-2 tests, logistic regression, multiple logistic regression, longitudinal analysis, AC2 with quadratic weights, risk ratios, odds ratios, 95% confidence intervals, and *p*-values ≤ 0.05 were considered statistically significant.

### Ethical considerations

All recommendations were in line with official Swedish SIDS and infant positioning guidelines that were in effect when the study was conducted [21]. Written informed parental consent was obtained. Intervention group nurses were asked to refer NSP cases that did not improve within two months to a physician, and to promptly consult a physician if craniosynostosis was suspected. Nurses in both groups were given the opportunity to contact the project leader if they had questions. If assessors deemed infants needed help for severe NSP, parents were offered appointments to the project leader for more in-depth advice. If an infant became upset or scared when the craniometer was used, cranial measurements were discontinued. When all data were collected, each control group nurse was offered the opportunity to participate in the nurse education at their own clinic. The Regional Ethical Review Board in Gothenburg approved the study (Dnr 418–11).

## Results

### Birth-related factors, side preference, and care factors

The two infant groups were, on the whole, similar regarding birth-related factors (Table 1). At two months, a smaller proportion of intervention group infants compared to control group infants were solely bottle-fed (21%; 35%, *p* = 0.01); and there was a wide range in parent-reported time infants in both groups spent in positional devices daily, most markedly the bouncer. The minimum-maximum time infants spent daily in a bouncer was zero minutes - eight hours in the intervention group, and zero minutes - 9 h 40 min in the control group.

**Table 1:** Birth-related factors, side preference, and infant care factors reported by intervention and control group parents

	Intervention group	Control group
	*n* = 176	*n* = 92
Birth-related factors		
male	95 (54%)	50 (54%)
birth weight (g)	3623 (2405–4870)	3638 (2425–5010)
gestational age (wks)	40 (36–43)	40 (35–43)
breach birth	1 (1%)	1 (1%)
vacuum-assisted delivery	20 (11%)	10 (11%)
firstborn	70 (40%)	38 (41%)
twin	6 (3%)	2 (2%)
born with a flat spot	8 (5%)	2 (2%)
Side preference at 2 months	78 (44%)	33 (36%)
Care factors at 2 months		
solely bottle-fed	36 (21%)	32 (35%)
estimated time spent daily (min.)		
in infant car seat	17 (0–240)	15 (0–150)
in infant bouncer	55 (0–480)	33 (0–580)
in stationary infant activity center	0 (0–270)	13 (0–180)
total daily time in positional devices (min.)	83 (0–480)	96 (0–590)
n (%) or medians (min-max)		

### Reliability

Using AC2, the inter-rater agreement between plagiocephaly assessment scores and CVAI measurement scores at T3 was 0.74 [0.68; 0.80]. The inter-rater agreement between brachycephaly assessment scores and CI measurement scores at T3 was 0.81 [0.78; 0.84]. When adjusted for chance agreement and interpreted, the size of both coefficients corresponds with substantial agreement, indicating that assessments made by trained assessors at T3 can be considered trustworthy when using the CVAI and CI measurements as the gold standard.

### The course of development

**Fig. 2:**
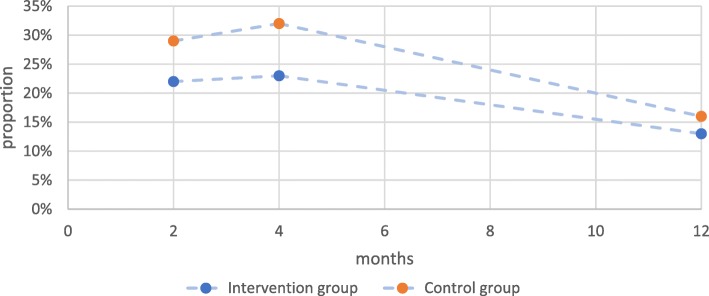
Proportions of intervention and control group infants at 2, 4, and 12 months with nonsynostotic plagiocephaly. Legend: The proportion of infants shown at each point includes new cases that developed and excludes cases that reversed since the preceding time, the net result of the ongoing processes of prevention and reversal

Figure 2 shows the proportion of infants in the two groups with NSP at 2-, 4- and 12-month assessments, i.e., at T1, T2 and T3. Each point is the net result of the ongoing processes of prevention and reversal. That is, at each point, the proportion shown includes new cases that developed and excludes cases that reversed since the preceding time.

**Fig. 3:**
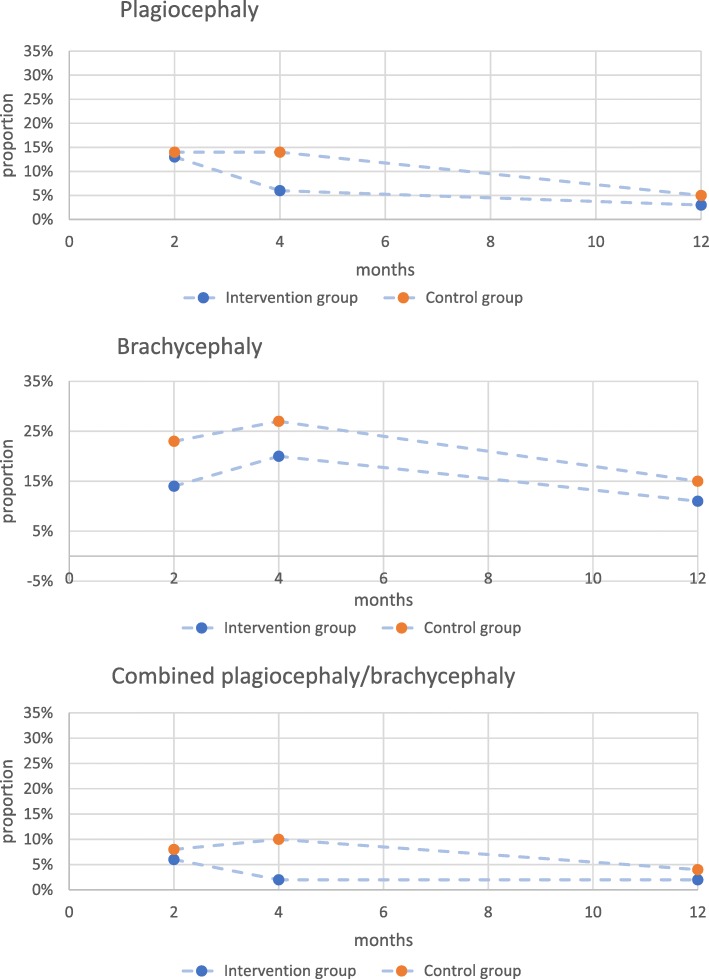
Course of development of 3 forms of nonsynostotic plagiocephaly in intervention and control group infants**.** Legend: The proportion of infants with brachycephaly increased between 2 and 4 months and then began to decrease in both groups. The proportion of intervention group infants with plagiocephaly began to decrease between 2 and 4 months and stayed low, while the proportion of control group infants with plagiocephaly began to decrease after 4 months. The proportion of intervention group infants with combined plagiocephaly/brachycephaly began to decrease between 2 and 4 months and stayed low, while the proportion of control group infants increased slightly between 2 and 4 months and began to decrease after 4 months

The proportion of infants with NSP was lower in the intervention group compared to the control group at all times. Still, the course of NSP development was similar in the two groups. The proportion of infants with NSP increased from T1 to T2, indicating more prevention failure than reversal. Then the proportion of infants with NSP decreased from T2 to T3, indicating there was now more reversal than prevention failure. From T1-T3, the proportion of infants in the intervention group decreased from 22 to 13% and the proportion of infants in the control group decreased from 29 to 16%. Likewise, the proportion of infants with brachycephaly increased between T1 and T2 and then decreased between T2 and T3 in both groups (Fig. 3). However, the course of plagiocephaly development was different in the two groups. In the intervention group, the proportion of infants with plagiocephaly began to decrease between T1 and T2 and continued to decrease between T2 and T3; while in the control group, the proportion of infants did not begin to decrease until after T2. The course of combined plagiocephaly/brachycephaly was different in the groups as well. The proportion of infants with combined plagiocephaly/brachycephaly began to decrease in the intervention group between T1 and T2 and then stayed at this level until T3, while the proportion of control group infants increased slightly between T1 and T2 and then decreased between T2 and T3. In a longitudinal analysis, intervention group infants with combined plagiocephaly/brachycephaly showed a significantly different course of development than control group infants (*p* = 0.04). These were the only statistically significant results in the net results.

### Prevention

The prevention effect was estimated by considering the opposite phenomenon to prevention, namely, the occurrence of prevention failures, i.e., when infants developed NSP and were considered cases (Table 2). Since measurements took place at three points in time, prevention failure was analyzed from the perspectives of early, late, and overall prevention failure, i.e. from T1-T3. Non-cases at the outset of each time was the starting point. We analyzed prevention failure of general NSP and the three main groups of NSP. No difference between groups was observed in general NSP prevention failure. Plagiocephaly prevention failure was significantly lower in the intervention group compared to the control group from T2-T3 (*p* = 0.04) and T1-T3 (*p* = 0.04), but numbers of cases were low. In contrast, brachycephaly prevention failure was higher in the intervention group compared to the control group from T2-T3 and T1-T3. However, these results are not statistically significant. In the subgroups of infants who were non-cases at T1, six of 138 (4%) intervention group and seven of 65 (11%) control group infants developed plagiocephaly, and 34 of 138 (25%) intervention group and 14 of 65 (22%) control group infants developed brachycephaly between T1 and T3. Thus, brachycephaly prevention failure was ≥ six times more common than plagiocephaly prevention failure in the intervention group.

**Table 2:** Prevention failure in intervention and control infants with no nonsynostotic plagiocephaly (NSP) at 2 months

	FAILED PREVENTION					Chi-2 test
	Intervention subgroups		Control subgroups			2- tailed
	Early prevention failure		Early prevention failure			
Cranial shape	non-cases T1 n	cases T2 n (%)	non-cases T1 n	cases T2 n (%)	RR	*p*-value
Plagiocephaly	138	6 (4)	65	5 (8)	1.75	0.33
Brachycephaly	138	25 (18)	65	12 (18)	1.02	0.95
Combination	138	3 (2)	65	4 (6)	2.86	0.15
NSP in total	138	28 (20)	65	13 (20)	.99	0.96
	Late prevention failure		Late prevention failure			
	non-cases T2 n	cases T3 n (%)	non-cases T2 n	cases T3 n (%)	RR	*p*-value
Plagiocephaly	132	0 (0)	60	2 (3)	0	0.04
Brachycephaly	113	9 (8)	53	2 (4)	0.47	0.31
Combination	135	0 (0)	61	1 (2)	0	0.14
NSP in total	110	9 (8)	52	3 (6)	0.70	0.58
	Overall prevention		Overall prevention			
	failure		failure			
	non-cases T1 n	cases T3 n (%)	non-cases T1 n	cases T3 n (%)	RR	*p*-value
Plagiocephaly	138	0 (0)	65	2 (3)	0	0.04
Brachycephaly	138	13 (9)	65	3 (5)	0.49	0.24
Combination	138	0 (0)	65	1 (2)	0	0.14
NSP in total	138	13 (9)	65	4 (6)	0.65	0.43

T1 = 2 months, T2 = 4 months, and T3 = 12 months

Combination = combined plagiocephaly/brachycephaly

RR = relative risk

Case = infant assessed with NSP

Non-case = infant did not have NSP at assessment

Prevention failure = infant developed NSP from one point in time to the next point in time

Infants who developed NSP after four months are considered “late developers” because the prevalence of NSP peaks at four months and then declines with time as the infant cranium becomes more ossified. Characteristics and care factors of infants where prevention failed after T2 were examined. Of the 12 late developers, 11 developed brachycephaly and four spent ≥ 3 h daily in positional devices at T1 (See Additional file 4: Table S2).

### Reversal

Reversal of general NSP and the three main groups of NSP was also analyzed from the perspectives of early, late, and overall reversal (Table 3). Cases at the outset of each time period were the starting point. There was a 24% difference between groups (65% intervention group; 41% control group) in NSP reduction from T1 to T2 (*p* = 0.06), considered early reversal. There was a 50% difference between groups (50% intervention group; 0% control group) in combined plagiocephaly/brachycephaly reduction from T1 to T2 (*p* = 0.03). Plagiocephaly and brachycephaly reduction from T1 to T2 pointed in the same direction, but these subgroups are small and results are not statistically significant. Nonetheless, these results also indicate that the intervention contributed to early reversal. There was a 10% difference between groups in NSP reduction from T2 to T3, indicating the intervention did not contribute much to late reversal. There was a 17% difference between groups in NSP reduction from T1 to T3, however, results are not statistically significant.

**Table 3:** Reversal in intervention and control infants with nonsynostotic plagiocephaly at 2 months and 4 months

	REVERSAL					Chi-2 test
	Intervention subgroups		Control subgroups			2 - sided
	Early reversal		Early reversal			
Cranial shape	cases T1 n	reversed by T2 n (%)	cases T1 n	reversed by T2 n (%)	RR	*p*-value
Plagiocephaly	23	.18 (78)	13	7 (54)	1.45	0.13
Brachycephaly*	25	16 (64)	21	9 (43)	1.49	0.15
Combination	10	.5 (50)	7	0 (0)	–	0.03
NSP in total	37	24 (65)	27	11 (41)	1.59	0.06
	Late reversal		Late reversal			
	cases T2 n	reversed by T3 n (%)	cases T2 n	reversed by T3 n (%)	RR	*p*-value
Plagiocephaly	10	8 (80)	13	10 (77)	1.04	0.86
Brachycephaly	35	27 (77)	25	16 (64)	1.21	0.27
Combination	4	4 (100)	9	6 (67)	1.50	0.19
NSP in total	41	31 (76)	29	19 (66)	1.15	0.36
	Overall reversal		Overall reversal			
	cases T1 n	reversed by T3 n (%)	cases T1 n	reversed by T3 n (%)	RR	*p*-value
Plagiocephaly	23	17 (74)	13	9 (69)	1.07	0.76
Brachycephaly	25	18 (72)	21	11 (52)	1.37	0.17
Combination	10	4 (40)	7	3 (43)	0.93	0.91
NSP in total	38	29 (76)	27	16 (59)	1.29	0.14

T1 = 2 months, T2 = 4 months, and T3 = 12 months

Combination = combined plagiocephaly/brachycephaly

NSP = nonsynostotic plagiocephaly

RR = relative risk

*= missing data for one infant

Case = infant assessed with NSP

**Fig. 4:**
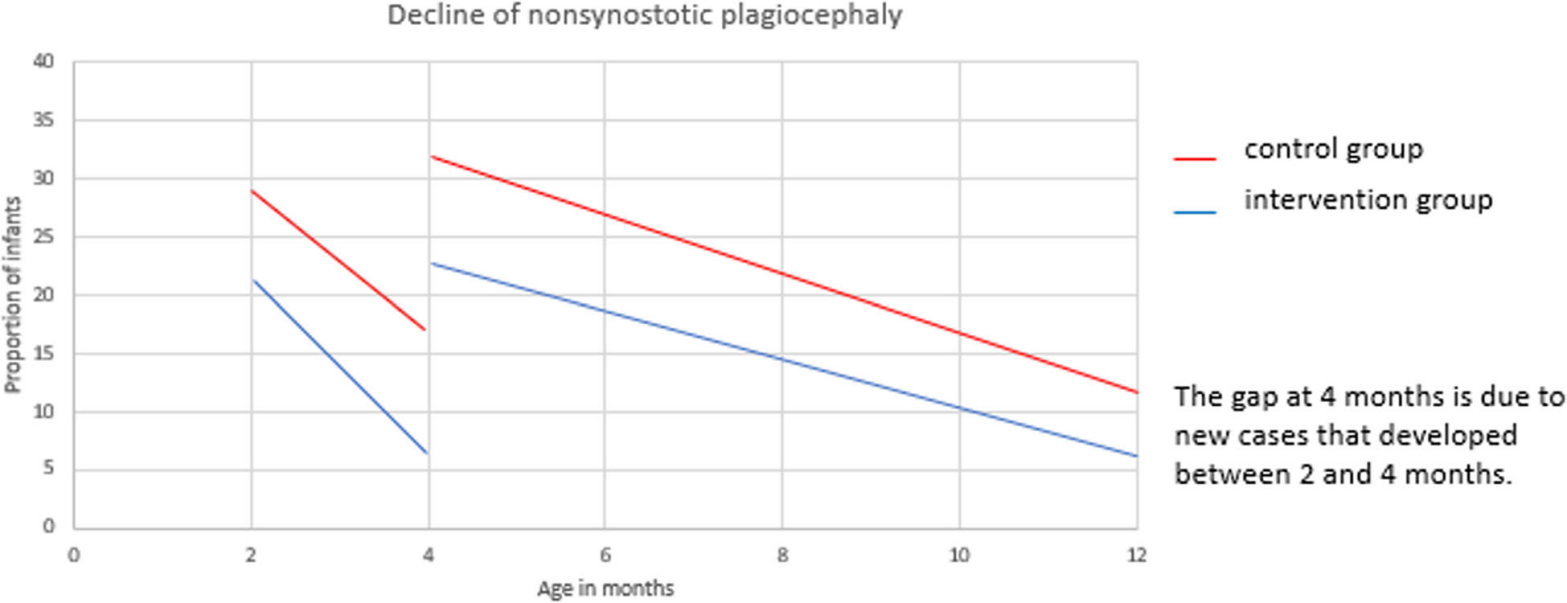
Decline of nonsynostotic plagiocephaly from 2 to 4 months and 4–12 months in intervention and control group infants**.** Legend: From 2 to 4 months, the decline in both slopes is steep. The gap in the slopes at 4 months is due to new cases that developed between 2 and 4 months. From 4 to 12 months, the decline in both slopes is less steep but more substantial. There was a decreased rate of reversal in both groups as infants grew older

The rate of reversal was analyzed in intervention and control group infants with NSP at the outset of T1 and T2. The decline of NSP from T1-T2 and from T2-T3 in the two groups is seen in Fig. 4. The gap in the slopes at four months is due to new cases that developed between T1 and T2. From T1-T2, the decline in both slopes is steep – a reduction of 14% in the intervention group and 12% in the control group within two months. From T2-T3, the decline in the slopes is less steep but more substantial – a reduction of 17% in the intervention group and 21% in the control group. Yet, when considering that the T2-T3 time span is four times longer than the T1-T2 time span, there was a decreased rate of reversal in both groups as infants grew older. At T3, 6% of intervention group infants and 11% of control group infants had NSP, however, the difference is not statistically significant.

Factors that might help explain reversal were investigated. It was four time more common that NSP at T1 reversed by T2 in intervention group than control group infants (OR = 4.07 [1.23; 13.44], *p* = 0.02) when adjusted for the effect of parent awareness of written information provided by their nurse at the first home visit. However, it was nine times more common that NSP at T1 reversed by T2 when parents were aware of written information from their nurse (OR = 9.09 [0.02; 0.48], *p* = 0.004) when adjusted for the effect of intervention group. Thus, it turned out that parent awareness of written information was more important in early reversal than the intervention itself. Moreover, it was > 5 times more common that NSP at T1 reversed by T3 when parents were aware of written information from their nurse (OR = 0.19 [0.04; 0.95], *p* = 0.04). This indicates that parent awareness of written information from their nurse was important in explaining reversal by T3 regardless of group.

### Infants who failed to reverse

Six (3%) intervention group and nine (10%) control group infants had NSP at each assessment, i.e. persistent asymmetry, (RR = 0.35 [0.13; 0.94], *p* = 0.03), indicating control group infants seemed to have nearly a threefold higher risk for persistent NSP at T3 than intervention group infants. Cranial shape course, birth-related factors, side preference, and care factors of these 15 infants were examined to understand what might hinder reversal of NSP (See Additional file 5: Table S3). Thirteen of the 15 infants had brachycephaly at T3. No single risk factor stood out, but 13 of the infants had two or more of the following risk factors: firstborn, side preference at T1, solely bottle-fed at T1, and spent ≥ 2 h daily in positional devices at T1.

To further investigate which risk factors might account for failure to reverse, birth-related factors, side preference, and care factors of these 15 infants were compared with those of infants whose NSP did reverse between T1 and T3 (Additional file 6: Table S4). A larger proportion of infants whose NSP failed to reverse were firstborn (60% vs 40%), had a vacuum-assisted delivery (27% vs 11%), and were solely bottle-fed at T1 (53% vs 33%). Moreover, their median daily bouncer time at T1 was higher (90 min vs 30 min). However, no significant differences were detected, and no conclusions can be drawn regarding associations between risk factors during early infancy and failure to reverse by 12 months.

## Discussion

The major findings of the study are the intervention was associated with early reversal and reduced an infant’s risk of having persistent NSP at 12 months. The results of agreement analysis indicate that the assessments conducted at T3 by trained assessors can be considered trustworthy. We believe this trustworthiness can be extended to assessments conducted at T1 and T2 as well, since these assessments were done in the same way.

We do not have a definitive baseline since molding from the birth process was reversing at the same time the intervention was underway. The influence of the intervention began six or seven weeks before we began collecting data because nurses in both groups made a home visit when newborns were about seven days old and gave the Child Health Book to parents in accordance with the national program. In addition, intervention group nurses were specifically encouraged to begin providing additional advice to parents at the first home visit, or at least by the time infants were two weeks old, because capitalizing on the plasticity of the neonatal cranium is the essence of intervention [22]. Data collection of cranial shape began when infants were two months old for three reasons. Firstly, NSP refers to acquired cranial asymmetry in infants older than six weeks of age when transient molding from the birth process - which can obscure true head shape - is over. Secondly, collecting data in conjunction with 2-month well-child visits facilitated planning logistics because some nurses could coordinate several 2-months visits on the same day for the assessors. This was helpful because infants’ appointments were spread out at 26 different child health clinics on different dates. Thirdly, we did not want to intrude on families by sending the assessors earlier. In short, there is no point in time that the intervention and control group infants were equal in prerequisites. However, we do know that infant groups were similar in regards to birth-related risk factors. Given this, neither infant group seemed to have an inherent disadvantage. Thus, although it is not possible to substantiate, the lower proportion of intervention group infants with NSP at two months compared to control group infants can be attributed – at least in part – to the intervention.

However, 38 (22%) of intervention group infants had NSP at two months. We had hoped that initiating strategies in the early neonatal period would have had a greater effect on prevention. We wondered if intervention group nurses had detected this NSP since no one initiates reversal efforts until asymmetry is identified. Intervention group nurses provided data on their 2-month cranial asymmetry assessments, so the nurses’ ability to detect cases was investigated by comparing the nurses’ and the assessors’ 2-month assessments. As it turned out, the intervention group nurses failed to detect 22 of 37 cases (59%) (missing data for one nurse). Moreover, we did a sensitivity analysis of the intervention group nurses’ 2-months assessments where assessors’ assessments were considered the gold standard. Intervention group nurses showed a 65% sensitivity (missing data from three nurses). Thus, nurses failing to detect nearly three of five cases in one explanation for the early prevention failure in the intervention group. Worth noting is that 16 of the 22 infants whose NSP nurses failed to detect at two months had reversed by four months and 17 had reversed by 12 months. Another explanation for the early prevention failure in the intervention group could be that some nurses were not sufficiently aware of risk factors. Early identification of infants with risk factors offers the best opportunity to prevent NSP [23]. According to Rogers, the most important risk factor to find out about is whether an infant has a head positional preference. Rogers recommends asking parents about this at the first well-child visit and evaluating the cervical range-of-motion with the neonate lying supine [23]. Intervention group nurses learned to evaluate the cervical range-of-motion in infants who were old enough to support their heads. On the other hand, perhaps early prevention failure was due to parent incompliance. Tummy time under surveillance is time consuming. Nurses could promote parent compliance by explicitly explaining the importance of tummy time under surveillance for head shape and infant safety, while at the same time conveying an understanding of how busy parents of newborns are.

Parent awareness precedes compliance. In our 4-month survey, the proportion of intervention group compared to control group parents that reported receiving verbal information from their nurse was 96% vs 85% respectively. However, the proportion of intervention group compared to control group parents that reported receiving written information from their nurse was only 58% vs 48% [13]. Plausible explanations for some parents’ unawareness of written information from their nurse include: information on NSP prevention was on page 38 in the Child Health Book and some nurses failed to point this out; parents of newborns are busy and some never read the information; parents are diverse regarding aptitude and some might not have understood the written information; and language difficulties. Language difficulties among immigrant parents potentially lowered the effect of our intervention. It would have been good if we had collected data on which parents had language difficulties, so that we could investigate how this influenced results. For recommendations to work, it is important to deliver them effectively. In a study evaluating a cultural and linguistically adapted parent education intervention involving Latinos, parents’ knowledge of tummy time was found to increase when educational materials were translated into Spanish and the intervention was presented by bilingual assistants [24].

We found that brachycephaly was more difficult to prevent than plagiocephaly in both groups. Some parents might consider central occipital flattening normal. Brachycephaly is considered a normal head shape in Asia where infants have traditionally been placed supine for sleep, and the head shape of American infants changed from normocephalic to brachycephalic less than a decade after the back-to-sleep campaign to prevent SIDS [25]. Infants with brachycephaly lay completely straight on their backs [18]. Once a cranial flat spot develops, it becomes an infant’s “comfort spot” and will be a hard habit to break [26]. Besides encouraging tummy time in early infancy whenever infants are awake, the nurse should point out early signs of brachycephaly to parents and explain that tummy time provides total occipital pressure relief.

Although not statistically significant, intervention group nurses and parents seemed to be more successful in reversing NSP at T1 by T2 than the control group (*p* = 0.06). We also found that it was four times more common that NSP at T1 reversed by T2 in intervention group than control group infants (OR = 4.07, *p* = 0.02) when adjusted for the effect of parent awareness of written information from their nurse. The intervention groups’ greater success in early reversal could be due to the intervention including specific reversal recommendations while the national recommendations do not. Another reason could be that intervention group nurses were taught how to assess cranial asymmetry, while control group nurses were not. However, we found that parents’ awareness of written information on NSP from their nurse was important in explaining reversal of NSP at T1 by T3 regardless of group. Parents were aware of written information from their nurse in 28 (62%) of the 45 cases that reversed by T3 (65% intervention group, 56% control group). This indicates that the prevention recommendations in the Child Health Book benefitted reversal as well, when parents were aware of them.

We were surprised that there were nine late developers in the intervention group. According to Rogers, it is rare to see progression of flattening after four months of age in term infants [23], and all were term infants, ± two weeks, except one who was born gestational week 37. All nine developed brachycephaly. We did not find any particular pattern regarding risk factors (Table S3). Six of the nine parents reported receiving written information from their nurse and eight reported receiving verbal information. We compared assessors’ and nurses’ cranial asymmetry assessments at T1 and T2. Assessors detected mild asymmetry in eight of the nine infants and only one nurse failed to detect this. Thus, in most cases of late development, there were early signs of incipient NSP which nurses detected. We cannot explain late development in the intervention group.

Based on the pilot study, we expected a greater effect of the intervention. Our sample size was based on the effect size of the pilot study when infants were six months old. However, we investigated effect size at other ages in this study. A larger sample would have resulted in better power to detect the effects displayed in this study. It is also possible that control group nurses and parents performed better than they would have otherwise since they were not only sensitized to NSP through participation in the study, but also alerted to NSP. It is unlikely that control group nurses’ and parents’ participation in the study did not influence them in any way.

The two previously mentioned studies with early intervention were designed differently than ours. In the prospective controlled study, brachycephaly was not included [8]. Plagiocephaly was diagnosed by a pediatrician determining skewness and recommendations were provided directly to parents at a strategic opportunity [18]. In contrast, we educated nurses to assess head shape and provide on-going tailored counseling to parents. The proportion of infants with plagiocephaly at four months in that study was 13% in the intervention group and 31% in the control group, while the proportions of infants with plagiocephaly in our study were 5 and 14% respectively. We seemed to do somewhat better by educating child health nurses to detect NSP with the Severity Assessments and guide parents in prevention and reversal strategies. However, the lower proportion of four-month-old infants with plagiocephaly in our study compared to theirs could be due to a favorable starting point due to Swedish national recommendations. Another possible explanation is that different cultures have different infant care practices. In our study, control group infants spent a mean of approximately two hours in positional devices daily according to parent-reported estimates, while control group infants in that study, a French study, spent a mean of approximately six hours in positional devices daily.

In the RCT evaluating early intervention, recommendations were provided directly to parents by a neonatologist in a 15-min private guidance session and in written form before discharge from the maternity unit; and NSP was assessed using 2 D and 3 D craniofacial imaging [9]. In that study, the prevalence of NSP was 11% in the intervention group and 31% in the control group in a 2 D analysis at *three* months. In our study, the prevalence of NSP was 23% in the intervention group and 32% in the control group at *four* months. This age difference is important when comparing net results because NSP peaks at about four months [6]. In their follow-up of the RCT investigating head shape of infants from three to 12 months, all parents concerned about their infant’s head shape received advice on repositioning, regardless of previous group allocation. When sorted according to original group allocation, 13% of intervention group infants and 20% of control group infants had NSP at 12 months [27]. In our study, 13% of intervention group and 16% of control group infants had NSP at 12 months. We seemed to do just as well by educating child health nurses to detect NSP with the Severity Assessments and guide parents in prevention and reversal strategies.

Since pillow recommendations were removed from the Swedish national recommendations in late 2013, our prevention strategies need more emphasis on occipital pressure relief when infants are awake. According to results in a cross-sectional survey, parents of children with NSP reported fewer minutes of estimated tummy time than parents of children who did not develop NSP (mean 26 min vs mean 49 min) [28]. It would have been useful if we had collected data on how much tummy time parents in our study provided for their infants.

Strengths of our study include: the prospective cohort design which was two-armed; assessors were reliability-tested prior to the intervention; the thorough analysis provided deeper knowledge regarding the effects of the intervention on prevention and reversal; 95% of nurses followed through; and 98% of infants and parents followed through. Limitations include: no data were collected on sociodemographic factors; and no data were collected on side preference and infant care factors after two months; the sample was too small for analyzing subgroups, so this was mostly exploratory; there was no validation of the scoring system; and changes in parents’ behavior and what nurses actually did were not addressed.

## Conclusions

The main contribution of the study is that we introduced the idea of analyzing prevention and reversal separately in order to more thoroughly evaluate the effects of the intervention. The intervention was associated with early reversal and reducing an infant’s risk of having persistent NSP at 12 months. However, brachycephaly was difficult to prevent in both groups. Results indicate that we are on the right track, yet nurses need to learn more about assessing NSP, and further research is needed in order to improve our strategies. We need to investigate ways to improve brachycephaly prevention. Since most parents were aware of verbal information from their nurse during the early months of infancy, we need to investigate how nurses can communicate recommendations more effectively. Moreover, since parent awareness of written information from their nurse had a significant effect on reversal, we need to investigate ways to promote a heightened awareness among parents of written recommendations from their nurse.

## Additional files

Additional file 1: **Figure S1.** CONSORT 2010 Flow Diagram 1. The nurses. (DOC 55 kb)

Additional file 2: **Figure S2.** CONSORT 2010 Flow Diagram 2. The infants. (DOC 53 kb)

Additional file 3: **Table S1.** Cranial asymmetry scores corresponding with cranial asymmetry indices for 10–12-month-old males and females*. (DOCX 12 kb)

Additional file 4: **Table S2.** Characteristics, care factors at T1, and head shape at T3 of “late developers”. (DOCX 14 kb)

Additional file 5: **Table S3.** Cranial shape and factors of infants whose nonsynostotic plagiocephaly at T1 failed to reverse by T3. (DOCX 15 kb)

Additional file 6: **Table S4.** Parent-reported factors in infants whose 2-month nonsynostotic plagiocephaly failed to reverse and reversed by 12 months. (DOCX 20 kb)

## Abbreviations

AC2: Agreement coefficient 2
CI: Cranial index
CVAI: Cranial vault asymmetry index
NSP: Nonsynostotic plagiocephaly
OR: Odds ratio
RCT: Randomized controlled trial
RR: Relative risk
SIDS: Sudden infant death syndrome
