# Supplementary material for: Nonsynostotic plagiocephaly: a child health care intervention in Skaraborg, Sweden
Source: BMC Pediatr. 2019 Feb 6;19:48. doi: 10.1186/s12887-019-1405-y (PMC6364473; doi:10.1186/s12887-019-1405-y)
Supplement: Supplementary file 1 — Figure S1. CONSORT 2010 Flow Diagram 1. The nurses. (DOC 55 kb) [file 12887_2019_1405_MOESM1_ESM.doc]

**
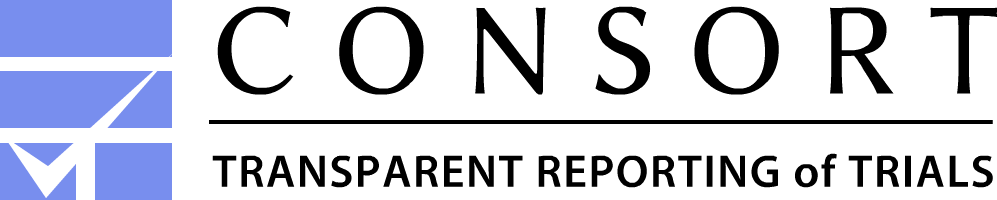
**

**CONSORT 2010 Flow Diagram**

**Allocation**

**Analysis**

**Follow-Up**

**Enrollment**

Eligible (n= 69)

Analysed (n= 35)
♦ Excluded from analysis (n= 3)

Lost to follow-up (n= 0)

Discontinued intervention (n= 0)

Analysed (n= 18)
♦ Excluded from analysis (n= 0)

1. **The nurses**

Excluded (n= 13)

♦  Declined to participate (n= 13)

Lost to follow-up (no longer employed) (n= 3)

Discontinued intervention (n = 0)

Allocated to intervention (n= 38)

♦ Participated in continuing education on cranial asymmetry (n= 38)

♦ Did not participate in continuing education (n=0)

Allocated to control (n= 18)

♦ Participated in continuing education on cranial asymmetry (n= 0)

♦ Did not participate in continuing education (n= 18)

Allocated to group (n= 56) 56)
