# Supplementary material for: Nonsynostotic plagiocephaly: a child health care intervention in Skaraborg, Sweden
Source: BMC Pediatr. 2019 Feb 6;19:48. doi: 10.1186/s12887-019-1405-y (PMC6364473; doi:10.1186/s12887-019-1405-y)
Supplement: Supplementary file 2 — Figure S2. CONSORT 2010 Flow Diagram 2. The infants. (DOC 53 kb) [file 12887_2019_1405_MOESM2_ESM.doc]

**
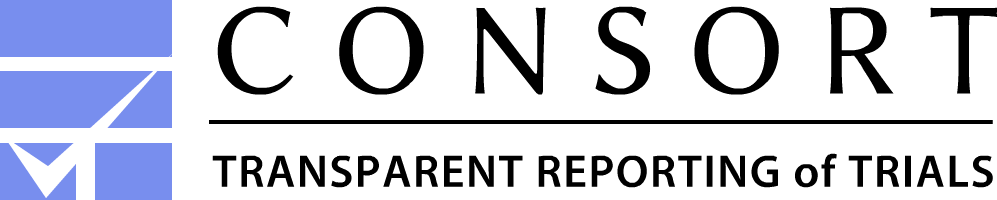
**

**CONSORT 2010 Flow Diagram**

**Allocation**

**Analysis**

**Follow-Up**

**Enrollment**

Eligible (n= 275)

Analysed (n= 176)
♦ Excluded from analysis (n= 6)

Lost to follow-up (Family moved) (n= 6)

Discontinued intervention (n= 0)

Allocated to intervention group (n= 182)

♦ Received intervention care (n= 182)

Lost to follow-up (n= 0)

Discontinued intervention (n= 0)

Analysed (n= 92)
♦ Excluded from analysis (n= 0)

Allocated (n= 274)

**2. The infants**

Excluded (n= 1)

♦  Not meeting inclusion criteria (n= 0)

♦  Parent declined to participate (n= 1)

Allocated to routine care group (n= 0)

♦ Received routine care (n= 92)
