# Supplementary material for: Nonsynostotic plagiocephaly: a child health care intervention in Skaraborg, Sweden
Source: BMC Pediatr. 2019 Feb 6;19:48. doi: 10.1186/s12887-019-1405-y (PMC6364473; doi:10.1186/s12887-019-1405-y)
Supplement: Supplementary file 3 — Table S1. Cranial asymmetry scores corresponding with cranial asymmetry indices for 10–12-month-old males and females*. (DOCX 12 kb) [file 12887_2019_1405_MOESM3_ESM.docx]

**Table S1. Cranial asymmetry scores corresponding with cranial asymmetry indices for 10-12-month-old males**

**and females***

| Asymmetry Score Cranial Vault Asymmetry Index Cranial Index  male female male female  none 0 < 2.8 < 3.1 < 86.4 < 85.8  mild 1 2.9 – 4.3 3.2 – 4.6 86.5 – 92.0 85.9 – 89.4  moderate 2 4.4 – 5.2 4.7 – 5.9 92.1 - 96.9 89.5 – 94.7  severe 3 > 5.3 > 6.0 > 97 > 94.8 |
| --- |

* = adapted from Wilbrand, 2012
