# Supplementary material for: Nonsynostotic plagiocephaly: a child health care intervention in Skaraborg, Sweden
Source: BMC Pediatr. 2019 Feb 6;19:48. doi: 10.1186/s12887-019-1405-y (PMC6364473; doi:10.1186/s12887-019-1405-y)
Supplement: Supplementary file 4 — Table S2. Characteristics, care factors at T1, and head shape at T3 of “late developers”. (DOCX 14 kb) [file 12887_2019_1405_MOESM4_ESM.docx]

**Table S2. Characteristics, care factors at T1, and head shape at T3 of “late developers”**

| INTERVENTION GROUP | | | | | |
| --- | --- | --- | --- | --- | --- |
|  |  |  | Min. daily in posi- | Min. daily in | Head shape at |
| Infant | Male | Side-preference * | tional devices at T1* | bouncer at T1* | T3 |
|  |  |  |  |  |  |
| A |  |  | 240 | 120 | brachycephaly |
| B |  |  | 80 | 60 | brachycephaly |
| C | x | x | 28 | 10 | brachycephaly |
| D | x |  | 73 | 30 | brachycephaly |
| E | x | x | 180 | 150 | brachycephaly |
| F | x |  | 11 | 0 | brachycephaly |
| G | x | x | 180 | 60 | brachycephaly |
| H | x | x | 90 | 60 | brachycephaly |
| I |  |  | 81 | 60 | brachycephaly |
|  |  |  |  |  |  |
| CONTROL GROUP | | | | | |
|  |  |  |  |  |  |
| J | x | x | 7 | 0 | brachycephaly |
| K |  | x | 118 | 90 | combination |
| L |  | x | 210 | 120 | plagiocephaly |
|  |  |  |  |  |  |

T1 = 2 months, T3 = 12 months

“Late developer” = infant who developed cranial asymmetry after 4 months

Combination = combined plagiocephaly/brachycephaly

* parent-reported
