# Supplementary material for: Nonsynostotic plagiocephaly: a child health care intervention in Skaraborg, Sweden
Source: BMC Pediatr. 2019 Feb 6;19:48. doi: 10.1186/s12887-019-1405-y (PMC6364473; doi:10.1186/s12887-019-1405-y)
Supplement: Supplementary file 5 — Table S3. Cranial shape and factors of infants whose nonsynostotic plagiocephaly at T1 failed to reverse by T3. (DOCX 15 kb) [file 12887_2019_1405_MOESM5_ESM.docx]

**Table S3. Cranial shape and factors of infants whose nonsynostotic plagiocephaly at T1 failed to reverse by T3**

| Intervention group | | | | | | |
| --- | --- | --- | --- | --- | --- | --- |
| Infant | Cranial shape  T1-T2-T3 | Birth-related factors* | Male | Side prefer- ence at T1 * | Solely bottle- fed at T1* | Min. daily time in posi-tional devices at T1*  (of which bouncer time) |
|  |  |  |  |  |  |  |
| A | P - P - P | GA 40 |  | x | x | 71 (60) |
| B | B - B - B | GA 41, firstborn, VAD | x |  | x | 93 (90) |
| C | C - B - P | GA 38, firstborn |  | x |  | 158 (120) |
| D | C - B - B | GA 38, firstborn | x | x | x | 67 (7) |
| E | C - B - B | GA 40, firstborn, 2875 g birth wt. | x | x | x | 210 (45) |
| F | C - P - C | GA 42, firstborn, VAD |  |  |  | 87 (10) |
|  |  |  |  |  |  |  |
| Control group | | | | | | |
|  |  |  |  |  |  |  |
| G | P - C - B | GA 39, firstborn |  | x | x | 120 (60) |
| H | B - B - B | GA 42, VAD | x |  |  | 26 (0) |
| I | B - B - B | GA 40, flat spot at birth |  |  | x | 17 (0) |
| J | B - B - B | GA 38 |  |  |  | 68 (60) |
| K | B - B - B | GA 39, firstborn |  |  |  | 45 (15) |
| L | C - B - B | GA 40 | x | x |  | 150 (30) |
| M | C - P - C | GA 41, firstborn, VAD |  | x |  | 120 (60) |
| N | C - C - C | GA 39 | x |  | x | 210 (150) |
| O | C - C - C | GA 40, firstborn |  | x | x | 285 (150) |
|  |  |  |  |  |  |  |

T1 = 2 months, T2 = 4 months, T3 = 12 months

B = brachycephaly; P = plagiocephaly; C = combined plagiocephaly/brachycephaly

GA= gestational age in weeks; VAD = vacuum-assisted delivery

* = parent-reported
