# Supplementary material for: Nonsynostotic plagiocephaly: a child health care intervention in Skaraborg, Sweden
Source: BMC Pediatr. 2019 Feb 6;19:48. doi: 10.1186/s12887-019-1405-y (PMC6364473; doi:10.1186/s12887-019-1405-y)
Supplement: Supplementary file 6 — Table S4. Parent-reported factors in infants whose 2-month nonsynostotic plagiocephaly failed to reverse and reversed by 12 months. (DOCX 20 kb) [file 12887_2019_1405_MOESM6_ESM.docx]

**Table S4. Parent-reported factors in infants whose 2-month nonsynostotic plagiocephaly failed to reverse and reversed by 12 months**

|  | Failed to reverse | | Reversed | |
| --- | --- | --- | --- | --- |
|  | n = 15 | | n = 45 | |
| Birth-related factors |  | |  | |
| male | 7 (46 %) | | 22 (49 %) | |
| birth weight (g) | 3538 (3035 - 4015) | | 3540 (2875 - 4600) | |
| gestational age (wks) | 40 (38 - 42) | | 40 (36 - 42) | |
| vacuum-assisted delivery | 4 (27 %) | | 5 (11%) | |
| first born | 9 (60 %) | | 20 (44 %) | |
| twin | 2 (2 %) | | 6 (3 %) | |
| born with a flat spot | 2 (13 %) | | 4 (8 %) | |
| Side preference at 2 months | 8 (53 %) | | 23 (51 %) | |
|  |  | |  | |
| Care factors at 2 months |  | |  | |
| solely bottle-fed | 8 (53 %) | | 15 (33%) | |
| estimated time spent daily (min.) |  | |  | |
| in infant car seat | 24 (08 - 60) | | 20 (0 - 120) | |
| in infant bouncer | 90 (10 - 150) | | 30 (0 - 360) | |
| in stationary infant activity center | 33 (0 - 90) | | 1 (0 - 270) | |
| total daily time in positional devices (min.) | 139 (71 - 285) | | 98 (15 - 373) | |
|  |  | |  | |
| n (%) or medians (min-max) | |  |  |  |
